# Tris(ethyl­enediamine-κ^2^ *N*,*N*′)cobalt(III) aqua­tris­(oxalato-κ^2^ *O* ^1^,*O* ^2^)indate(III)

**DOI:** 10.1107/S1600536811053736

**Published:** 2011-12-17

**Authors:** Zhe Zhang, Fuxiang Wang, Shuangquan Liao

**Affiliations:** a Department of Materials and Chemical Engineering, Ministry of Education Key Laboratory of Application Technology of Hainan Superior Resources Chemical Materials, Hainan University, Haikou 570228, Hainan Province, People’s Republic of China

## Abstract

In the cation of the title compound, [Co(C_2_H_8_N_2_)_3_][In(C_2_O_4_)_3_(H_2_O)], the Co^III^ atom is coordinated by six N atoms from three ethyl­enediamine mol­ecules. The Co^III^—N bond lengths lie in the range 1.956 (4)–1.986 (4) Å. In the anion, the In^III^ atom is seven-coordinated by six O atoms from three oxalate ligands and by a water mol­ecule. The cations and anions are linked by extensive O—H⋯O and N—H⋯O hydrogen bonds, forming a supermolecular network.

## Related literature

For metal phosphates and germanates templated by metal complexes, see: Du *et al.* (2004 ▶); Pan *et al.* (2005 ▶, 2008 ▶); Wang *et al.* (2003*a* ▶,*b* ▶,*c* ▶). For coordination polymers templated by metal complexes, see: Pan *et al.* (2010*a* ▶,*b* ▶, 2011 ▶), Tong & Pan (2011 ▶). 
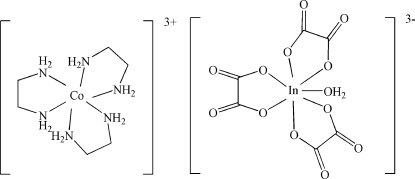


## Experimental

### 

#### Crystal data

[Co(C_2_H_8_N_2_)_3_][In(C_2_O_4_)_3_(H_2_O)]
*M* *_r_* = 636.14
Triclinic, 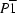

*a* = 7.5161 (15) Å
*b* = 10.921 (2) Å
*c* = 14.450 (3) Å
α = 79.43 (3)°
β = 80.13 (3)°
γ = 71.25 (3)°
*V* = 1096.1 (4) Å^3^
*Z* = 2
Mo *K*α radiation
μ = 1.89 mm^−1^
*T* = 293 K
0.3 × 0.2 × 0.18 mm

#### Data collection

Rigaku R-AXIS RAPID-S diffractometer
Absorption correction: multi-scan (*CrystalClear*; Rigaku/MSC, 2002 ▶) *T*_min_ = 0.6, *T*_max_ = 0.8
11056 measured reflections
4988 independent reflections
4360 reflections with *I* > 2σ(*I*)
*R*_int_ = 0.056

#### Refinement

*R*[*F* ^2^ > 2σ(*F* ^2^)] = 0.048
*wR*(*F* ^2^) = 0.119
*S* = 1.10
4988 reflections
300 parameters
Only H-atom displacement parameters refined
Δρ_max_ = 1.32 e Å^−3^
Δρ_min_ = −0.91 e Å^−3^

### 

Data collection: *RAPID-AUTO* (Rigaku, 1998 ▶); cell refinement: *RAPID-AUTO*; data reduction: *CrystalStructure* (Rigaku/MSC, 2002 ▶); program(s) used to solve structure: *SHELXS97* (Sheldrick, 2008 ▶); program(s) used to refine structure: *SHELXL97* (Sheldrick, 2008 ▶); molecular graphics: *SHELXTL* (Sheldrick, 2008 ▶); software used to prepare material for publication: *SHELXTL*.

## Supplementary Material

Crystal structure: contains datablock(s) I, global. DOI: 10.1107/S1600536811053736/fj2490sup1.cif

Structure factors: contains datablock(s) I. DOI: 10.1107/S1600536811053736/fj2490Isup2.hkl

Additional supplementary materials:  crystallographic information; 3D view; checkCIF report

## Figures and Tables

**Table 1:** Hydrogen-bond geometry (Å, °)

*D*—H⋯*A*	*D*—H	H⋯*A*	*D*⋯*A*	*D*—H⋯*A*
O13—H13*A*⋯O12^i^	0.91	1.79	2.620 (5)	150
O13—H13*B*⋯O6^ii^	0.86	1.84	2.629 (4)	152
N1—H1*A*⋯O11^iii^	0.90	2.17	3.064 (5)	171
N1—H1*B*⋯O8^iv^	0.90	2.14	2.972 (5)	153
N2—H2*A*⋯O7^v^	0.90	2.10	2.935 (5)	155
N2—H2*B*⋯O10^vi^	0.90	2.01	2.838 (5)	152
N3—H3*A*⋯O7^v^	0.90	2.34	3.142 (5)	149
N3—H3*A*⋯O3^v^	0.90	2.37	3.063 (5)	134
N3—H3*B*⋯O6^iv^	0.90	2.06	2.878 (5)	151
N4—H4*A*⋯O1^iii^	0.90	2.26	3.114 (5)	159
N4—H4*A*⋯O2^iii^	0.90	2.49	3.103 (5)	126
N4—H4*B*⋯O2	0.90	2.04	2.924 (6)	168
N5—H5*A*⋯O8^v^	0.90	2.09	2.962 (5)	163
N5—H5*B*⋯O4	0.90	2.14	2.912 (5)	143
N5—H5*B*⋯O2	0.90	2.40	3.099 (5)	135
N6—H6*A*⋯O11^iii^	0.90	2.27	3.040 (5)	143
N6—H6*A*⋯O1^iii^	0.90	2.49	3.279 (5)	147
N6—H6*B*⋯O10^vi^	0.90	2.31	3.105 (5)	147

Symmetry codes: (i) 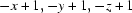; (ii) 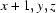; (iii) 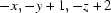; (iv) 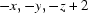; (v) 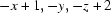; (vi) 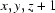.

**Fig. 1.**
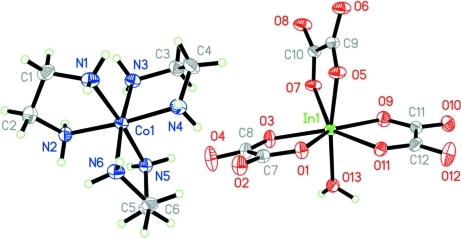
A view of the structure of complex. Ellipsoids are drawn at the 30% probability level.

## supplementary crystallographic information

### Comment

Currently, more attention has been paid to employ chiral metal complexes as template, for its wide of shapes, charges and particularly chirality. Up to now, series of metal phosphates and germanates with interesting stuctures have been prepared by using such chiral metal complexes as the template (Wang *et al.*, 2003*a*; Pan *et al.* 2005, 2008; Du *et al.* 2004). And a new concept of chirality transfer of the metal complex into the inorganic host framework has been demonstrated by Yu *et al.*(Wang *et al.*, 2003*b*,*c*). Recently, Pan *et al.* introduced it into the system coordination polymers, a series of metal oxalates were obtained using metal complex cations as template (Pan *et al.*, 2010*a*,*b*, 2011). More recently, they reported [Co(C_2_H_8_N_2_)_3_]^3+^[In(C_2_O_4_)_2_(CHO_2_)_2_]^3-^ .2H_2_O, a formate oxalate mixed coordinated complex (Tong & Pan 2011). In this paper, we present a new complex [Co(C_2_H_8_N_2_)_3_]^3+^[In(C_2_O_4_)_3_(H_2_O)]^3-^. As shown in Fig. 1, the crystal structure of (I) consists of a discrete [In(C_2_O_4_)_3_(H_2_O)]^3-^ anions and [Co(en)_3_]^3+^ cations. The In^III^ ions was seven coordinated and surrounded by three different chelting oxalate and a coordinated water molecule. And the Co^III^ atom in the cation was connected six N atoms from three different chelting ethylenediamine in a distorted octahedral geometry. The cations and the anions were connected each other through hydrogen bonds to giving a supermolecule entity.

### Experimental

In a typical synthesis, a mixture of In(NO_3_)_3_.5H_2_O (1 mmol), Co(en)_3_Cl_3_ (0.14 mmol), K_2_C_2_O_4_.H_2_O (2 mmol) and H_2_O (10 ml), was added to a 20 ml Teflon-lined reactor under autogenous pressure at 120 °C for 4 days.

### Refinement

All H atoms were positioned geometrically (C—H = 0.97 Å, N—H = 0.90 Å and O—H = 0.85 Å) and allowed to ride on their parent atoms, with *U*_iso_(H) = 1.2*U*_eq_(parent atom).

### Crystal data

**Table:**

[Co(C_2_H_8_N_2_)_3_][In(C_2_O_4_)_3_(H_2_O)]	*Z* = 2
*M**_r_* = 636.14	*F*(000) = 640
Triclinic, *P*1	*D*_x_ = 1.927 Mg m^−^^3^
*a* = 7.5161 (15) Å	Mo *K*α radiation, λ = 0.71073 Å
*b* = 10.921 (2) Å	Cell parameters from 10592 reflections
*c* = 14.450 (3) Å	θ = 3.0–27.5°
α = 79.43 (3)°	µ = 1.89 mm^−^^1^
β = 80.13 (3)°	*T* = 293 K
γ = 71.25 (3)°	Block, yellow
*V* = 1096.1 (4) Å^3^	0.3 × 0.2 × 0.18 mm

### Data collection

**Table:**

Rigaku R-AXIS RAPID-S diffractometer	4988 independent reflections
Radiation source: fine-focus sealed tube	4360 reflections with *I* > 2σ(*I*)
graphite	*R*_int_ = 0.056
ω scans	θ_max_ = 27.5°, θ_min_ = 3.0°
Absorption correction: multi-scan (*CrystalClear*; Rigaku/MSC, 2002)	*h* = −9→9
*T*_min_ = 0.6, *T*_max_ = 0.8	*k* = −14→14
11056 measured reflections	*l* = −18→18

### Refinement

**Table:**

Refinement on *F*^2^	Primary atom site location: structure-invariant direct methods
Least-squares matrix: full	Secondary atom site location: difference Fourier map
*R*[*F*^2^ > 2σ(*F*^2^)] = 0.048	Hydrogen site location: inferred from neighbouring sites
*wR*(*F*^2^) = 0.119	Only H-atom displacement parameters refined
*S* = 1.10	*w* = 1/[σ^2^(*F*_o_^2^) + (0.0504*P*)^2^ + 0.7829*P*] where *P* = (*F*_o_^2^ + 2*F*_c_^2^)/3
4988 reflections	(Δ/σ)_max_ = 0.001
300 parameters	Δρ_max_ = 1.32 e Å^−^^3^
0 restraints	Δρ_min_ = −0.91 e Å^−^^3^

### Special details

**Table:**

Geometry. All e.s.d.'s (except the e.s.d. in the dihedral angle between two l.s. planes) are estimated using the full covariance matrix. The cell e.s.d.'s are taken into account individually in the estimation of e.s.d.'s in distances, angles and torsion angles; correlations between e.s.d.'s in cell parameters are only used when they are defined by crystal symmetry. An approximate (isotropic) treatment of cell e.s.d.'s is used for estimating e.s.d.'s involving l.s. planes.
Refinement. Refinement of *F*^2^ against ALL reflections. The weighted *R*-factor *wR* and goodness of fit *S* are based on *F*^2^, conventional *R*-factors *R* are based on *F*, with *F* set to zero for negative *F*^2^. The threshold expression of *F*^2^ > σ(*F*^2^) is used only for calculating *R*-factors(gt) *etc*. and is not relevant to the choice of reflections for refinement. *R*-factors based on *F*^2^ are statistically about twice as large as those based on *F*, and *R*- factors based on ALL data will be even larger.

### Fractional atomic coordinates and isotropic or equivalent isotropic displacement parameters (Å^2^)

**Table:**

	*x*	*y*	*z*	*U*_iso_*/*U*_eq_	
In1	0.41280 (4)	0.27178 (3)	0.70832 (2)	0.02767 (11)	
Co1	0.13688 (7)	0.26192 (5)	1.23880 (4)	0.02715 (15)	
O1	0.2854 (5)	0.4022 (3)	0.8190 (2)	0.0379 (7)	
O2	0.2698 (5)	0.4142 (3)	0.9718 (2)	0.0490 (9)	
O3	0.5453 (5)	0.1704 (3)	0.8361 (2)	0.0402 (8)	
O4	0.5603 (6)	0.1847 (4)	0.9864 (3)	0.0681 (12)	
O5	0.1303 (4)	0.2508 (3)	0.7207 (3)	0.0400 (8)	
O6	−0.0236 (4)	0.1146 (3)	0.7043 (3)	0.0480 (9)	
O7	0.4574 (4)	0.0573 (3)	0.7116 (2)	0.0386 (7)	
O8	0.2982 (5)	−0.0880 (3)	0.7345 (3)	0.0517 (9)	
O9	0.4602 (5)	0.2478 (3)	0.5559 (2)	0.0449 (8)	
O10	0.4079 (6)	0.3539 (4)	0.4111 (2)	0.0564 (10)	
O11	0.2819 (4)	0.4654 (3)	0.6321 (2)	0.0349 (7)	
O12	0.2215 (6)	0.5763 (4)	0.4920 (3)	0.0660 (12)	
O13	0.6771 (4)	0.3184 (3)	0.6787 (2)	0.0382 (7)	
H13A	0.690 (3)	0.380 (4)	0.628 (3)	0.080*	
H13B	0.792 (5)	0.272 (2)	0.686 (3)	0.080*	
N1	−0.1017 (6)	0.2554 (4)	1.3206 (3)	0.0410 (9)	
H1A	−0.1676	0.3355	1.3353	0.080*	
H1B	−0.1734	0.2288	1.2894	0.080*	
N2	0.2587 (5)	0.1761 (4)	1.3530 (3)	0.0353 (8)	
H2A	0.3232	0.0924	1.3464	0.080*	
H2B	0.3414	0.2163	1.3607	0.080*	
N3	0.1680 (5)	0.0943 (3)	1.1973 (3)	0.0362 (8)	
H3A	0.2883	0.0433	1.2001	0.080*	
H3B	0.0917	0.0534	1.2364	0.080*	
N4	0.0045 (6)	0.3375 (4)	1.1268 (3)	0.0394 (9)	
H4A	−0.1014	0.4015	1.1420	0.080*	
H4B	0.0790	0.3727	1.0815	0.080*	
N5	0.3809 (5)	0.2696 (3)	1.1686 (3)	0.0334 (8)	
H5A	0.4736	0.2015	1.1922	0.080*	
H5B	0.3818	0.2628	1.1074	0.080*	
N6	0.1154 (5)	0.4369 (4)	1.2661 (3)	0.0376 (9)	
H6A	−0.0062	0.4868	1.2690	0.080*	
H6B	0.1564	0.4305	1.3223	0.080*	
C1	−0.0561 (8)	0.1636 (5)	1.4084 (4)	0.0488 (12)	
H1C	−0.0303	0.0745	1.3967	0.080*	
H1D	−0.1620	0.1822	1.4579	0.080*	
C2	0.1141 (8)	0.1807 (5)	1.4380 (4)	0.0489 (12)	
H2C	0.0817	0.2637	1.4616	0.080*	
H2D	0.1630	0.1115	1.4881	0.080*	
C3	0.1212 (8)	0.1146 (5)	1.0987 (4)	0.0470 (12)	
H3C	0.0886	0.0404	1.0866	0.080*	
H3D	0.2283	0.1253	1.0534	0.080*	
C4	−0.0453 (8)	0.2366 (5)	1.0901 (4)	0.0490 (13)	
H4C	−0.0684	0.2643	1.0244	0.080*	
H4D	−0.1584	0.2208	1.1269	0.080*	
C5	0.4190 (7)	0.3938 (4)	1.1746 (3)	0.0388 (10)	
H5C	0.4919	0.4199	1.1165	0.080*	
H5D	0.4909	0.3819	1.2270	0.080*	
C6	0.2320 (7)	0.4966 (4)	1.1895 (3)	0.0384 (10)	
H6C	0.2491	0.5728	1.2078	0.080*	
H6D	0.1718	0.5228	1.1318	0.080*	
C7	0.3368 (6)	0.3589 (4)	0.9016 (3)	0.0349 (10)	
C8	0.4958 (7)	0.2256 (4)	0.9099 (3)	0.0396 (10)	
C9	0.1204 (6)	0.1394 (4)	0.7148 (3)	0.0345 (10)	
C10	0.3067 (6)	0.0259 (4)	0.7209 (3)	0.0347 (10)	
C11	0.3953 (7)	0.3486 (5)	0.4982 (3)	0.0392 (11)	
C12	0.2895 (6)	0.4741 (4)	0.5431 (3)	0.0380 (10)	

### Atomic displacement parameters (Å^2^)

**Table:**

	*U*^11^	*U*^22^	*U*^33^	*U*^12^	*U*^13^	*U*^23^
In1	0.02796 (18)	0.02283 (16)	0.03195 (19)	−0.00503 (12)	−0.00555 (12)	−0.00598 (11)
Co1	0.0254 (3)	0.0220 (3)	0.0337 (3)	−0.0050 (2)	−0.0045 (2)	−0.0058 (2)
O1	0.0447 (19)	0.0308 (15)	0.0320 (17)	0.0010 (13)	−0.0064 (14)	−0.0096 (13)
O2	0.063 (2)	0.0385 (18)	0.0382 (19)	−0.0011 (16)	−0.0030 (16)	−0.0148 (15)
O3	0.049 (2)	0.0324 (16)	0.0285 (17)	0.0043 (14)	−0.0057 (14)	−0.0049 (13)
O4	0.084 (3)	0.064 (2)	0.035 (2)	0.017 (2)	−0.021 (2)	−0.0108 (17)
O5	0.0311 (16)	0.0246 (15)	0.064 (2)	−0.0069 (13)	−0.0073 (15)	−0.0074 (14)
O6	0.0311 (17)	0.0337 (17)	0.081 (3)	−0.0111 (14)	−0.0150 (17)	−0.0010 (16)
O7	0.0299 (16)	0.0235 (14)	0.064 (2)	−0.0061 (12)	−0.0103 (15)	−0.0092 (14)
O8	0.045 (2)	0.0237 (15)	0.090 (3)	−0.0096 (14)	−0.0235 (19)	−0.0037 (16)
O9	0.062 (2)	0.0368 (17)	0.0375 (19)	−0.0119 (16)	−0.0079 (16)	−0.0119 (14)
O10	0.088 (3)	0.064 (2)	0.0312 (19)	−0.037 (2)	−0.0131 (18)	−0.0094 (16)
O11	0.0371 (17)	0.0289 (15)	0.0360 (18)	−0.0072 (13)	−0.0048 (13)	−0.0018 (12)
O12	0.074 (3)	0.054 (2)	0.045 (2)	0.003 (2)	−0.001 (2)	0.0100 (18)
O13	0.0284 (16)	0.0433 (18)	0.0405 (19)	−0.0103 (14)	−0.0089 (14)	0.0036 (14)
N1	0.037 (2)	0.036 (2)	0.051 (3)	−0.0107 (17)	−0.0003 (18)	−0.0150 (18)
N2	0.034 (2)	0.038 (2)	0.032 (2)	−0.0093 (16)	−0.0043 (16)	−0.0031 (15)
N3	0.035 (2)	0.0281 (18)	0.045 (2)	−0.0071 (16)	−0.0074 (17)	−0.0065 (16)
N4	0.037 (2)	0.0297 (19)	0.051 (2)	−0.0055 (16)	−0.0143 (18)	−0.0031 (17)
N5	0.034 (2)	0.0295 (18)	0.034 (2)	−0.0058 (15)	−0.0048 (16)	−0.0040 (15)
N6	0.035 (2)	0.0336 (19)	0.045 (2)	−0.0090 (16)	−0.0006 (17)	−0.0140 (16)
C1	0.053 (3)	0.050 (3)	0.046 (3)	−0.025 (3)	0.013 (2)	−0.014 (2)
C2	0.053 (3)	0.058 (3)	0.037 (3)	−0.021 (3)	0.001 (2)	−0.009 (2)
C3	0.065 (3)	0.041 (3)	0.042 (3)	−0.020 (2)	−0.013 (2)	−0.011 (2)
C4	0.054 (3)	0.046 (3)	0.055 (3)	−0.016 (2)	−0.026 (3)	−0.006 (2)
C5	0.040 (3)	0.039 (2)	0.043 (3)	−0.018 (2)	−0.002 (2)	−0.010 (2)
C6	0.047 (3)	0.027 (2)	0.045 (3)	−0.016 (2)	−0.008 (2)	−0.0044 (19)
C7	0.035 (2)	0.028 (2)	0.040 (3)	−0.0077 (18)	−0.0037 (19)	−0.0062 (18)
C8	0.042 (3)	0.038 (2)	0.034 (3)	−0.006 (2)	−0.006 (2)	−0.0024 (19)
C9	0.029 (2)	0.029 (2)	0.045 (3)	−0.0074 (18)	−0.0074 (19)	−0.0020 (18)
C10	0.037 (2)	0.025 (2)	0.044 (3)	−0.0088 (18)	−0.014 (2)	−0.0025 (18)
C11	0.048 (3)	0.045 (3)	0.033 (3)	−0.024 (2)	−0.009 (2)	−0.005 (2)
C12	0.034 (2)	0.037 (2)	0.040 (3)	−0.0125 (19)	−0.002 (2)	0.0031 (19)

### Geometric parameters (Å, °)

**Table:**

In1—O13	2.160 (3)	N2—H2B	0.9000
In1—O5	2.182 (3)	N3—C3	1.487 (6)
In1—O3	2.191 (3)	N3—H3A	0.9000
In1—O11	2.199 (3)	N3—H3B	0.9000
In1—O9	2.221 (3)	N4—C4	1.477 (6)
In1—O1	2.222 (3)	N4—H4A	0.9000
In1—O7	2.250 (3)	N4—H4B	0.9000
Co1—N4	1.956 (4)	N5—C5	1.494 (5)
Co1—N5	1.957 (4)	N5—H5A	0.9000
Co1—N2	1.959 (4)	N5—H5B	0.9000
Co1—N3	1.960 (4)	N6—C6	1.479 (6)
Co1—N6	1.972 (4)	N6—H6A	0.9000
Co1—N1	1.986 (4)	N6—H6B	0.9000
O1—C7	1.277 (5)	C1—C2	1.492 (7)
O2—C7	1.225 (5)	C1—H1C	0.9700
O3—C8	1.263 (5)	C1—H1D	0.9700
O4—C8	1.234 (6)	C2—H2C	0.9700
O5—C9	1.262 (5)	C2—H2D	0.9700
O6—C9	1.236 (5)	C3—C4	1.509 (7)
O7—C10	1.265 (5)	C3—H3C	0.9700
O8—C10	1.244 (5)	C3—H3D	0.9700
O9—C11	1.266 (6)	C4—H4C	0.9700
O10—C11	1.238 (6)	C4—H4D	0.9700
O11—C12	1.265 (5)	C5—C6	1.501 (6)
O12—C12	1.230 (6)	C5—H5C	0.9700
O13—H13A	0.9135	C5—H5D	0.9700
O13—H13B	0.8610	C6—H6C	0.9700
N1—C1	1.481 (7)	C6—H6D	0.9700
N1—H1A	0.9000	C7—C8	1.559 (6)
N1—H1B	0.9000	C9—C10	1.546 (6)
N2—C2	1.491 (6)	C11—C12	1.543 (7)
N2—H2A	0.9000		
			
O13—In1—O5	170.86 (11)	H4A—N4—H4B	108.1
O13—In1—O3	79.09 (12)	C5—N5—Co1	111.8 (3)
O5—In1—O3	109.59 (13)	C5—N5—H5A	109.3
O13—In1—O11	87.46 (12)	Co1—N5—H5A	109.3
O5—In1—O11	83.75 (12)	C5—N5—H5B	109.3
O3—In1—O11	143.84 (12)	Co1—N5—H5B	109.3
O13—In1—O9	84.67 (13)	H5A—N5—H5B	107.9
O5—In1—O9	90.49 (13)	C6—N6—Co1	108.4 (3)
O3—In1—O9	136.66 (12)	C6—N6—H6A	110.0
O11—In1—O9	73.94 (12)	Co1—N6—H6A	110.0
O13—In1—O1	95.45 (12)	C6—N6—H6B	110.0
O5—In1—O1	84.56 (12)	Co1—N6—H6B	110.0
O3—In1—O1	73.95 (11)	H6A—N6—H6B	108.4
O11—In1—O1	74.14 (11)	N1—C1—C2	107.7 (4)
O9—In1—O1	148.04 (12)	N1—C1—H1C	110.2
O13—In1—O7	111.82 (12)	C2—C1—H1C	110.2
O5—In1—O7	74.38 (11)	N1—C1—H1D	110.2
O3—In1—O7	72.79 (12)	C2—C1—H1D	110.2
O11—In1—O7	142.94 (12)	H1C—C1—H1D	108.5
O9—In1—O7	76.66 (12)	N2—C2—C1	107.3 (4)
O1—In1—O7	131.28 (12)	N2—C2—H2C	110.2
N4—Co1—N5	92.52 (17)	C1—C2—H2C	110.2
N4—Co1—N2	175.52 (16)	N2—C2—H2D	110.2
N5—Co1—N2	90.62 (16)	C1—C2—H2D	110.2
N4—Co1—N3	84.54 (16)	H2C—C2—H2D	108.5
N5—Co1—N3	91.28 (16)	N3—C3—C4	106.4 (4)
N2—Co1—N3	92.20 (16)	N3—C3—H3C	110.5
N4—Co1—N6	91.28 (16)	C4—C3—H3C	110.5
N5—Co1—N6	84.23 (15)	N3—C3—H3D	110.5
N2—Co1—N6	92.22 (16)	C4—C3—H3D	110.5
N3—Co1—N6	173.73 (16)	H3C—C3—H3D	108.6
N4—Co1—N1	92.14 (18)	N4—C4—C3	106.5 (4)
N5—Co1—N1	174.76 (16)	N4—C4—H4C	110.4
N2—Co1—N1	84.87 (17)	C3—C4—H4C	110.4
N3—Co1—N1	91.56 (16)	N4—C4—H4D	110.4
N6—Co1—N1	93.26 (16)	C3—C4—H4D	110.4
C7—O1—In1	116.6 (3)	H4C—C4—H4D	108.6
C8—O3—In1	118.2 (3)	N5—C5—C6	107.9 (4)
C9—O5—In1	117.0 (3)	N5—C5—H5C	110.1
C10—O7—In1	114.6 (3)	C6—C5—H5C	110.1
C11—O9—In1	116.8 (3)	N5—C5—H5D	110.1
C12—O11—In1	116.9 (3)	C6—C5—H5D	110.1
In1—O13—H13A	118.1	H5C—C5—H5D	108.4
In1—O13—H13B	132.1	N6—C6—C5	106.2 (4)
H13A—O13—H13B	104.0	N6—C6—H6C	110.5
C1—N1—Co1	109.3 (3)	C5—C6—H6C	110.5
C1—N1—H1A	109.8	N6—C6—H6D	110.5
Co1—N1—H1A	109.8	C5—C6—H6D	110.5
C1—N1—H1B	109.8	H6C—C6—H6D	108.7
Co1—N1—H1B	109.8	O2—C7—O1	124.7 (4)
H1A—N1—H1B	108.3	O2—C7—C8	119.9 (4)
C2—N2—Co1	110.4 (3)	O1—C7—C8	115.4 (4)
C2—N2—H2A	109.6	O4—C8—O3	126.7 (4)
Co1—N2—H2A	109.6	O4—C8—C7	117.8 (4)
C2—N2—H2B	109.6	O3—C8—C7	115.6 (4)
Co1—N2—H2B	109.6	O6—C9—O5	125.6 (4)
H2A—N2—H2B	108.1	O6—C9—C10	118.4 (4)
C3—N3—Co1	110.6 (3)	O5—C9—C10	116.0 (4)
C3—N3—H3A	109.5	O8—C10—O7	125.0 (4)
Co1—N3—H3A	109.5	O8—C10—C9	118.4 (4)
C3—N3—H3B	109.5	O7—C10—C9	116.5 (4)
Co1—N3—H3B	109.5	O10—C11—O9	125.9 (5)
H3A—N3—H3B	108.1	O10—C11—C12	118.6 (4)
C4—N4—Co1	110.6 (3)	O9—C11—C12	115.5 (4)
C4—N4—H4A	109.5	O12—C12—O11	123.6 (5)
Co1—N4—H4A	109.5	O12—C12—C11	119.6 (4)
C4—N4—H4B	109.5	O11—C12—C11	116.8 (4)
Co1—N4—H4B	109.5		

### Hydrogen-bond geometry (Å, °)

**Table:**

*D*—H···*A*	*D*—H	H···*A*	*D*···*A*	*D*—H···*A*
O13—H13A···O12^i^	0.91	1.79	2.620 (5)	150
O13—H13B···O6^ii^	0.86	1.84	2.629 (4)	152
N1—H1A···O11^iii^	0.90	2.17	3.064 (5)	171
N1—H1B···O8^iv^	0.90	2.14	2.972 (5)	153
N2—H2A···O7^v^	0.90	2.10	2.935 (5)	155
N2—H2B···O10^vi^	0.90	2.01	2.838 (5)	152
N3—H3A···O7^v^	0.90	2.34	3.142 (5)	149
N3—H3A···O3^v^	0.90	2.37	3.063 (5)	134
N3—H3B···O6^iv^	0.90	2.06	2.878 (5)	151
N4—H4A···O1^iii^	0.90	2.26	3.114 (5)	159
N4—H4A···O2^iii^	0.90	2.49	3.103 (5)	126
N4—H4B···O2	0.90	2.04	2.924 (6)	168
N5—H5A···O8^v^	0.90	2.09	2.962 (5)	163
N5—H5B···O4	0.90	2.14	2.912 (5)	143
N5—H5B···O2	0.90	2.40	3.099 (5)	135
N6—H6A···O11^iii^	0.90	2.27	3.040 (5)	143
N6—H6A···O1^iii^	0.90	2.49	3.279 (5)	147
N6—H6B···O10^vi^	0.90	2.31	3.105 (5)	147

Symmetry codes: (i) −*x*+1, −*y*+1, −*z*+1; (ii) *x*+1, *y*, *z*; (iii) −*x*, −*y*+1, −*z*+2; (iv) −*x*, −*y*, −*z*+2; (v) −*x*+1, −*y*, −*z*+2; (vi) *x*, *y*, *z*+1.
